# Conscious processing of auditory regularities induces a pupil dilation

**DOI:** 10.1038/s41598-018-33202-7

**Published:** 2018-10-04

**Authors:** Marion Quirins, Clémence Marois, Mélanie Valente, Magali Seassau, Nicolas Weiss, Imen El Karoui, Jean-Rémy Hochmann, Lionel Naccache

**Affiliations:** 1 INSERM, U 1127, F-75013 Paris, France; 2 Institut du Cerveau et de la Moelle épinière, ICM, PICNIC Lab, F-75013 Paris, France; 3 AP-HP, Groupe hospitalier Pitié-Salpêtrière-Charles Foix, Department of Neurology, Paris, France; 4 AP-HP, Groupe hospitalier Pitié-Salpêtrière-Charles Foix, Department of Neurophysiology, Paris, France; 5 Sorbonne Universités, UPMC Univ Paris 06, Faculté de Médecine Pitié-Salpêtrière, Paris, France; 6 INSERM UMR_S 938, CDR Saint-Antoine Maladies métaboliques, biliaires et fibro-inflammatoires du foie, Paris, France; 7 Institut de Cardiométabolisme et Nutrition, ICAN, Paris, France; 8 Institut de neurosciences translationnelles IHU-A-ICM, Paris, France; 9 CNRS, UMR 5304, Institut des Sciences Cognitives Marc Jeannerod, Bron, France; 10 Université Claude Bernard Lyon 1, Lyon, France

## Abstract

Pupil dilation has been reliably identified as a physiological marker of consciously reportable mental effort. This classical finding raises the question of whether or not pupil dilation could be a specific somatic signature of conscious processing. In order to explore this possibility, we engaged healthy volunteers in the ‘local global’ auditory paradigm we previously designed to disentangle conscious from non-conscious processing of novelty. We discovered that consciously reported violations of global (inter-trials) regularity were associated with a pupil dilation effect both in an active counting task and in a passive attentive task. This pupil dilation effect was detectable both at the group-level and at the individual level. In contrast, unreported violations of this global regularity, as well as unreported violations of local (intra-trial) regularity that do not require conscious access, were not associated with a pupil dilation effect. We replicated these findings in a phonemic version of the ‘local global’. Taken together these results strongly suggest that pupil dilation is a somatic marker of conscious access in the auditory modality, and that it could therefore be used to easily probe conscious processing at the individual level without interfering with participant’s stream of consciousness by questioning him/her.

## Introduction

For decades, pupil size has been reliably reported as a physiological marker of mental effort (see in particular the seminal review in Chapter 2 of^1^) corresponding to a combination of both a parasympathetic pathway inhibition (inhibition of the contractor muscle) and of a sympathetic pathway activation (dilation driven by high-level cognitive processes from perception to decision-making^2^). Several studies revealed the impact of high-level cognitive processes on pupil diameter^3^. For instance, Naber and colleagues designed an original pupil-frequency tagging approach to discover that endogenous allocation of visual attention induces a pupil dilation effect^4^. Similarly, mental imagery of brightness^5^, mind-wandering^6^ and consciously reported musical^7,8^ and poetry^9^ aesthetic feelings affect pupil diameter. This literature leads to the interesting hypothesis that pupil dilation could be a somatic behavioral signature of conscious access. In support of this hypothesis, Wessel *et al*.^10^ observed a larger pupil dilation effect for consciously perceived errors as compared to unreported errors in an anti-saccade task. Against this hypothesis, Diede and colleagues recently adapted the flanker task so to present mostly compatible trials at a specific screen location, and mostly incompatible trials at another location. They reported a pupil dilation effect in response to compatibility proportion whereas participants were unable to report any difference of compatibility proportion between the two locations^11^. However, the dissociation between pupil dilation and awareness was obtained by probing participants’ awareness at the experiment level and not at the single trial-level. Awareness of compatibility proportion may therefore have occurred on a few trials, and may have triggered a conscious executive control effect transferred to following trials and operant even in the absence of awareness on following trials^12^. In any case, regarding pupil dilation as a somatic marker of conscious access remains an interesting hypothesis that requires additional tests to be validated or refuted.

If confirmed, such a discovery could be used as a somatic proxy to conscious access, much simpler and less constraining than current functional brain imaging tools (e.g.: fMRI, M/EEG). This could pave the way to original paradigms enabling the detection of conscious access to various representations, without interfering with participants’ stream of consciousness by questioning them, further enabling the study of consciousness in individuals unable to respond, such as non-verbal infants (e.g.,^13,14^) and non-human primates^15^. Finally, such a somatic signature of conscious access could be used at bedside in non-communicating patients, - such as patients in a vegetative state (VS) or in a minimally conscious state (MCS) -, as recently illustrated in a proof of concept study^16^. However, to date we are still lacking a direct and univocal evidence linking conscious reports during a perceptual task with pupil dilation.

In the present study, we tested this hypothesis by engaging conscious controls in a pupillometry version of the ‘local global’ auditory paradigm we previously designed to disentangle conscious processing of inter-trials global regularity from non-conscious processing of intra-trials local regularity^17^. Our prediction was two-fold: first, global novelty should be associated with a pupil dilation effect while local effect should not, and second, this global effect should depend on conscious access to global regularity. We used a tonal and a phonemic version of this local global paradigm in two distinct labs. Our results confirmed the first prediction, and provided strong evidence supporting that this pupil dilation effect could be a marker of conscious access to global regularity.

## Materials and Methods

In this study, we performed four experiments conducted in two independent laboratories (Experiments 1 and 2 in Paris, and 3 and 4 in Lyon) in a total of 127 healthy adult volunteers. While the pupillometry devices were different, we used a same general approach. Studies in different labs were initially planned and run independently, which explains local differences in the analysis of the data. However, the strong reproducibility of our results across the two sites reinforces their strength and validity.

### Pupillometry

Blinks are physiological movements than can, at least partially, be voluntary controlled. The ability to control them varies widely from one subject to the next. Therefore, one key methodological issue was related to the processing of trials artefacted with eye-blinks or eye-movements. In the first experiment we deliberately used two approaches and compared them: (1) a trial rejection approach that minimizes the risk of interpreting artefactual signal as genuine pupillometry data, but that also exposes to a drastic reduction of conserved trials and therefore of statistical power; and (2) an interpolation approach that aims at correcting eye-blinks rather than rejecting blinked trials. Thus, this interpolation approach enables to increase the number of valid trials but exposes to the risk of using more noisy data. Experiment 1 showed that both methods enabled us to obtain very similar results. As a consequence, we moved to the interpolation method for the remaining 3 experiments.

### Statistics

We used the same rigorous methodology in each of the 4 experiments, with slight differences across the two experimental sites (Paris and Lyon). More precisely, we applied a non-parametric permutation-based procedure, - also coined mass-cluster test -, initially designed for EEG/MEG datasets^18^ that we previously used for pupillometry^13^, and that evaluates the significance of temporal event-related pupil diameter (ERPD) clusters and corrects for multiple comparisons. Namely, we first applied a first-level parametric student t-test with a double criterion (p-value and minimal number of successive samples satisfying this p-value criterion). For each significant cluster identified with this first-level test, we computed the sum of absolute value of t-values. We then created surrogate datasets by assigning randomly each trial to a condition, and controlling that the original number of trials per condition was preserved. These surrogate datasets were then submitted to the first-level procedure described above. We repeated this permutation procedure N times (N = 5000 in Paris, and N = 1000 in Lyon) times. We could then estimate the α–risk or the probability of observing randomly each of the clusters identified on genuine datasets. We required each cluster to satisfy a p-value ≤ 0.05 on this second-level statistics correcting for multiple comparisons. The very same approach was used both for group-level and individual statistics. The only difference between our two laboratories dealt with the number of permutations (N = 5000 for Paris, and N = 1000 for Lyon), and with the first-level thresholds (p ≤ 0.01 for a minimum of 160 ms in Paris, and p ≤ 0.05 and no minimum duration in Lyon) that only acted here as a first-level procedure. Note that each of the Paris clusters were kept by replicating the procedure with the N = 1000 used in Lyon, and that each of the Lyon clusters were kept by replicating the procedure with the N = 5000 used in Paris. Note also that the more or less conservative threshold selected for the first-level statistics only acted as a preselection procedure that was then corrected at the second-level stage with a p-value identical across the two experimental sites. The value of the threshold does not modify the rate of false positive results^18^.

## Experiments 1 and 2

All experiments were approved by the local ethical committee (Comité de protection des personnes, Ile de France I), all participants gave a written informed consent and all the different investigations were conducted according to the principles expressed in the Declaration of Helsinki.

### Participants

We recruited 63 volunteers (mean age = 29.7 ± 10.1 years old; sex-ratio = 0.63) between August 2013 and October 2014. Inclusion criteria were age (≥18 years old), social security affiliation and French spoken language. Exclusion criteria were pregnancy and relevant medical history or ophthalmologic disorder other than minor refractive errors.

### Pupillometry data acquisition and analysis

Pupillometric data were recorded with the Mobile EBT^®^ eyetracked (e(ye)BRAIN, www.eye-brain.com, France) that received CE marking approval for medical purposes. The Mobile EBT1 benefits from a high frequency camera that allows it to record both the horizontal and vertical eye positions independently and simultaneously for each eye at a 120 Hz sampling rate. Participants lied down in decubitus 30° in a quiet room, and luminosity conditions were adjusted for each participant in order to optimize pupil detection. A calibration was performed before each experimental block to ensure quality of pupil detection. We deliberately choose not to control for luminance intensity to fit the experimental conditions we could encounter with patients affected with disorder of consciousness and hospitalized in intensive care units or related clinical environments. Auditory stimuli were delivered by the MeyeParadigm^®^ software and raw data were stored and extracted by MeyeAnalysis^®^ software (www.eye-brain.com, France). Analyses were performed using Matlab 7.0^®^ scripts (Mathworks, Natick, MA, USA, http://www.mathworks.com).

#### Blink management

As explained above, two different methods were used separately to deal with blinks artefacts in this first experiment. The first method consisted in detecting blinks through subtraction of successive pupillometry values. Given that, unlike blinks, pupil dilation and constriction are continuous phenomenon, blinks were defined by a subtraction value greater than 200 pixels. Data were then segmented in trials and labelled according to their local and global regularity (standard or deviant). One trial started at the beginning of the first sound and ended 3000 ms later. Trials containing one or more blink were excluded from the analysis. No other signal processing was done in the remaining trials.

In the second method we used, blinks were detected via the (horizontal diameter [DX]/vertical diameter [DY]) ratio. We defined a blink when this DX/DY ratio, was out of [average ± standard deviation] interval (calculated over the whole block). Once a blink was detected, pupillometry values were estimated by a cubic interpolation. Interpolated blocks were then checked, and discarded when too noisy (i.e.>25% data being interpolated or infinite interpolated values). After interpolation, elementary trials were segmented as previously described.

### Auditory paradigm and procedure

To probe conscious processing, we previously designed an auditory local-global paradigm to study ERPs in healthy controls and in non-communicating patients^17,19–21^. Here, we adapted this paradigm to the slower dynamics of pupillometry.

**Figure 1:**
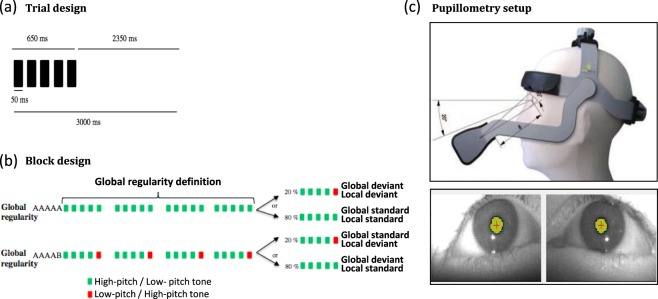
Auditory paradigm and pupillometry setup (**a**) On each trial 5 sounds were presented. (**b**) Each experimental block started with series of sounds defining the global regularity, before delivering global standard (80%) or global deviant (20%) trials. (**c**) EBT®-mobile device including two independent infrared camera centered on each pupil captures pupil images of each eye at a sampling rate of 120 Hz.

This auditory paradigm manipulates orthogonally two types of auditory regularities (Fig. 1). Repeated series of five sounds (SOA 150 ms) were presented via the laptop speakers. Each sound lasted 50 ms and could be either a low-pitched tone (A) or a high-pitched tone (B). Four different series were used, the first two using the same five sounds and conserving the local regularity (either AAAAA or BBBBB, called local standard stimulus); and the other two with the final sound swapped to break the local regularity (either AAAAB or BBBBA, called local deviant stimulus). Global regularity was defined by the repetition of one of the series in about 80% of the trials (global standard or frequent stimulus). This global regularity was broken in about 20% of the trials per block by presentation of an alternative series with a different local regularity (global deviant or infrequent stimulus). Finally, we used four block types by experiment designed by the frequent sequence (global standard): type AAAAA (AAAAA frequent/AAAAB infrequent), type BBBBB (BBBBB frequent/BBBBA infrequent), AAAAB (AAAAB frequent/AAAAA infrequent), BBBBA (BBBBA frequent/BBBBB infrequent). Each block started with five identical series of the frequent type to define the global regularity. The number of infrequent stimuli per block was randomized between 4 and 7 and each one was followed by a frequent stimulus to remind the global regularity. The time interval between two series was 3000 ms (instead of 1350–1650ms in evoked potentials) in order to adapt to the slower dynamics of pupil dilation/constriction. Each block included 30 trials, and so lasted 90 seconds.

#### Experiment 1: Active counting version of the local-global task

Instructions were auditory delivered at the beginning of each block as follow: “You will now listen to repetitive series of 5 sounds. At the beginning, series are all the same and define’the rule’. In a second time, some series will be different from the first ones. When you will hear such a series, different from the rule, we ask you to pay attention to it and to count it in your head. We will ask you at the end of the block how many different series you have counted.” Participants were stimulated with each of the four different block types according to a fixed order (AAAAA, AAAAB, BBBBB, BBBBA).

#### Experiment 2: Passive attentive version of the local-global task

In order to determine if pupil dilation depended upon awareness of the auditory rule or upon active counting of global deviant trials, we ran a “passive attentive” version of the task during which 38 participants were simply instructed to listen attentively to the sounds with no further explanation. A post-experimental interview was then proposed to score the Regularity Awareness Score we previously designed^17^. More precisely, Participants were first asked to comment on the experiment (“*Can you describe what you heard?”*). Free reports were collected and followed, when necessary, by a series of explicit questions about items that were not spontaneously reported. These questions were the following: Q1 “Is there any regularity in the sounds?”, Q2 “Are there any series of tones different than others across blocks?”, Q3 “Do the different series of sounds start immediately in each block?”, Q4 “Is there a pattern or rhythm between the infrequent stimuli?”, Q5 “Have you found infrequent stimuli in the form AAAAB or BBBBA?”, Q6 “Have you found infrequent stimuli in the form AAAAA or BBBBB?”.

In a second phase, these participants performed the classical local-global task with instructions.

### Statistics

A 200 ms baseline correction was applied. We then used a two-stage process to test for the presence of significant differences between conditions. Note that the local effect contrasts local standard trials (LS = AAAAA & BBBBB) to local deviant trials (LD = AAAAB and BBBBA), while the global effect contrasts frequent trials (GS for global standard) to infrequent trials (GD for global deviant) orthogonally to the local regularity.

#### Group analyses

For each participant, we computed the mean ERPD for each of the four conditions of interest (LD, LS, GD, GS). We then ran a t-test with a double-threshold condition: an effect was considered as possible only if the p value was ≤ 0.01 on 20 successive samples (160 ms). For each significant cluster on this first-level statistics, we computed the sum of the absolute values of t-values. Then, in order to take into account the multiple comparison problem, we ran a permutation test with surrogate data. We randomized the N × 2 individual mean vectors (e.g.: 60GS, 60GD) in two surrogate distributions. For each of such permutations (5000 permutations) we then ran the double-threshold procedure, and computed the sum of absolute values for each significant cluster. This allowed us to compute an alpha risk by calculating the proportion of surrogate clusters superior or equal to the observed sum of absolute values of t-values. We considered an effect as significant if this proportion was ≤0.05. This method is particularly relevant to estimate the statistical significance of effects observed with a signal of unknown distribution^22^.

#### Individual analyses

We used the very same approach to compute individual statistics. Single-trial data replaced mean ERPD data (see immediately above: Group analyses paragraph).

## Experiments 3 and 4

The procedure was adapted from the paradigm designed by Hochmann and Papeo^13^ to test infants. Participants were tested at the Institut des Sciences Cognitives Marc Jeannerod in Bron. All experiments were approved by the local ethical committee (Comité de protection des personnes, Sud-Est II), all participants gave a written informed consent and all the different investigations were conducted according to the principles expressed in the Declaration of Helsinki.

### Participants

Sixteen adult participants (18 to 30-year-olds; mean age = 22.9 ± 2.2 years; sex-ratio = 0.87) were recruited for Experiment 3 and 48 (18 to 30-year-olds; mean age = 21.7 ± 2.3years; sex-ratio = 0.65) for Experiment 4. All participants reported normal vision.

### Stimuli

Two syllables, /ba/ and /di/, were created with the artificial speech synthetizer MBROLA (French voice database FR4), with phoneme duration of 120 ms and pitch of 200 Hz. Each syllable was normalized to an intensity of 70 dB. Four sequences were created: ba ba ba ba, di di di di, ba ba ba di and di di di ba. The onset of two consecutive syllables was separated by 400 ms. Soundtracks were combined with a video clip lasting 4420 ms and showing a smiling cartoon character jumping repeatedly (www.GoAnimate.com).

### Pupillometry data acquisition and analysis

Fixations were identified by PsyScope X following the dwell-time algorithm^23^ with the following paramenters: WindowLength = 200, MinFixationLength = 100, DistanceFromMean = 0.05. We defined an area of interest (660 pi x 432 pi) corresponding to the surface of the video played on the screen to attract participants’ gaze. The pupil diameter for both eyes was recorded for fixations in that area of interest. For each trial, we considered a baseline time window beginning 500 ms before the onset of the last syllable of the sequence. The average pupil diameter in the baseline window was subtracted from all data points.

We analyzed the variation of the pupil diameter in a time window of interest starting with the beginning of the baseline window and ending with the trial (−500 ms to 3000).

Trials for which pupil diameter information was missing for more than the 25% of the time window of interest were marked as “bad trials” and excluded from further analyses. Ten percent of all trials were marked as bad trials in Experiment 3, and 14% were marked as bad trials in Experiment 4. Missing data for good trials were linearly interpolated.

### Auditory paradigm and procedure

In Experiment 3, participants were instructed at the beginning of the first block that they were going to hear one repeated sequence of syllables. They were asked to count the number of times that sequence changed. The same instructions were repeated at the beginning of the second block. In Experiment 4, participants were solely asked to pay attention to the auditory stimuli.

Participants sat in front of a Tobii eyetracker T60XL. Stimuli presentation and eyetracking data recording were controlled by PsyScope XL. Gaze and pupil data were acquired at a rate of 60 Hz. All lights in the room were switched off, except for that coming from the eyetracker’s screen. Participants took two blocks (Block 1: GS = LS; Block 2: GS = LD) containing each 100 trials (76 standard trials and 24 deviant trials). The order of blocks was randomized between participants. A calibration was performed before each block to ensure quality of pupil detection. Each trial was initiated by participants when they looked at a central fixation cross on the screen. The order of trial types was randomized, except for the first 4 trials that were always GS and were not analyzed.

For half of the participants, one block had ba ba ba ba (GS and LS) as global standard sequence and ba ba ba di (GD and LD) as global deviant sequence, and the other block had di di di ba (GS and LD) as global standard sequence and di di di di (GD and LS) as global deviant sequence. For the other half of the participants, one block had di di di di (GS and LS) as global standard sequence and di di di ba (GD and LD) as global deviant sequence, and the other block had ba ba ba di (GS and LD) as global standard sequence and ba ba ba ba (GD and LS) as global deviant sequence.

### Statistics

#### Group Analyses

Global and Local effects were defined as in Experiments 1 and 2. For the group analysis, we first computed the mean ERPD for each condition and for each participant. We then computed paired t-tests at each time point for the local and global effects. We identified clusters of adjacent time points with t-value larger than 2.05 ( ≤ p 0.05). For each cluster, we computed the cluster statistics as the sum of absolute t-values. Finally, in order to take into account the multiple comparison problem, we ran a permutation test (1000 permutations). For each permutation, we identified clusters and computed their summary statistics as on the original data. Finally, we computed the proportion of surrogate effects superior or equal to the observed cluster statistics in the original data. We considered an effect as significant if this proportion was ≤ 0.05.

#### Individual analyses

The same approach was used to compute single participant statistics, except that single-trial data replaced mean ERPD data and an unpaired Welch’s t-test was used to define clusters.

## Results

### Experiment 1

#### Behavior

The majority of the 60 participants counted the exact number of global deviant trials in each of the four conditions (median error-rate = 0; mean error-rate = 0.069 ± 0.2). This large standard deviation was explained by two participants who showed an unusual large level of errors during the first experimental block. One of them counted the number of GS trials rather than the number of GD (T16), while the other (T07) counted the whole number of trials delivered during this block. After discarding these 2 blocks that corresponded to an incorrect use of the instructions, we could confirm the almost perfect performance of counting GD trials (median error rate = 0; mean error-rate = 0.041 ± 0.09). Note that group analyses of pupil diameters were not affected by the inclusion/exclusion of these two blocks. Behavioral performance did not vary across the 4 conditions (F(3,236) = 1.1, p = 0.35).

#### Pupillometry

**Figure 2:**
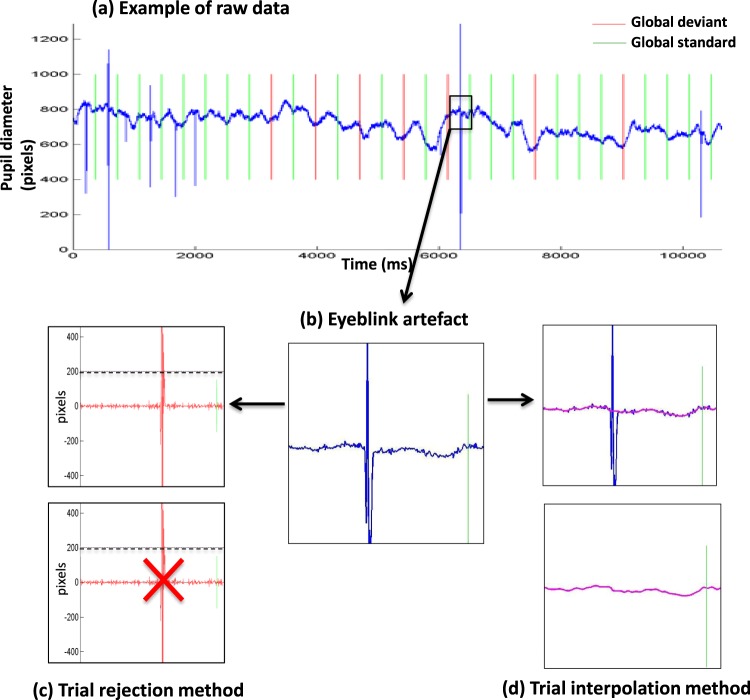
Raw data and two methods to process eye-blink artefacts (**a**) Example of raw data (blue curve): temporal evolution of pupil horizontal diameter (in pixels) from the right eye of one participant, superimposed with global standard trials in green and global deviant trials in red. (**b**) Zoom on a trial with an eye blink artefact processed with two different methods. (**c**) With the trial rejection method a trial was rejected if the difference between two successive temporal points was larger than 200 pixels. (**d**) With the trial interpolation method (see details in the text) any identified artefact (blue) was corrected using a cubic interpolation (bottom magenta curve).

**Figure 3:**
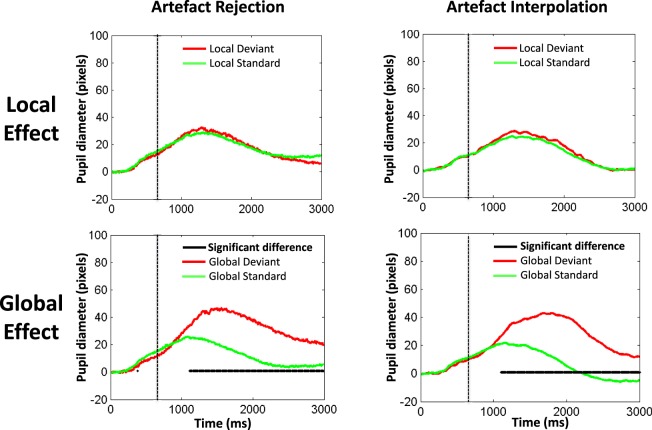
A pupil dilation occurred during violations of global regularity. Grand-averages of right eye pupil horizontal diameter (in pixels) are plotted over time (in milliseconds) in the active counting version of the ‘local global’ task both for the local (upper panels) and global (lower panels) effects. The artefact rejection (N = 56 participants; left panels) and artefact interpolation (N = 60 participants; right panels) methods led to the same results. While the event-related pupil response was undistinguishable between local standard (green curves) and local deviant (red curves) trials, a strong global effect was observed: a significant (black horizontal segments) pupil dilation was observed in response to global deviant trials. This effect began around 1 second after trial onset, peaked around 1.5 second and was sustained during more than 3 seconds. This effect was observed on horizontal and vertical diameters of both eyes. The vertical dotted line indicates the temporal offset of the five sounds defining a trial.

At the group level, both the rejection (N = 56, see below for details) and the interpolation (N = 60) methods led to similar results. While a clear event-related pupil dilation (ERPD) was observed in response to auditory trials, this response was not modulated by the local effect. In sharp contrast, a significant larger pupil dilation was observed after global deviant trials than after global standard ones (see Figs 2 and 3a). Given that ERPD responses were very similar in the 4 measured diameters (horizontal/vertical X left/right eye diameters; see Figures S1 and S2), we then restricted our analyses to left eye horizontal diameter data, discarding the possibility that our results could be subject to measurement errors and biases related to left/right or horizontal/vertical asymmetries in corneal reflection^24^. Inclusion or exclusion of the two controls who did not perform the instructed task (see above) did not change these two results.

At the individual level, the interpolation method enabled to analyze 60 participants, whereas only 56 could be explored with the rejection method due to too many blinks: all the trials of at least one category (GS, GD, LS and/or LD) were rejected in 4 participants. The interpolation method revealed that a significant global effect (pupil dilation to GD trials) was present in 36/60 (60% with a permutation corrected p-value ≤ 0.05), and we could observe a trend of this effect (0.05 < p ≤ 0.15) in 9 more participants (75%). In sharp contrast, only 2 participants (3%) showed a local effect, one presenting as a larger pupil dilation to LD than to LS trials, while the second one showed a reverse pattern.

### Experiment 2

In order to check if a pupil dilation in response to GD could be observed in absence of active counting task instructions, we recruited 38 control participants who were stimulated with the very same set of stimuli as in Experiment 1 in a passive attentive version of the task as used in a previous study^25^. After completing this passive attentive version of the task, we then recorded them in the active counting version of the local global paradigm, and included them in Experiment 1 dataset. Note that both behavior and ERPD results of Experiment 1 did not differ between this subgroup (N = 38) and the remaining 22 participants who performed the active counting task only (Welch test p value = 0.9).

**Figure 4:**
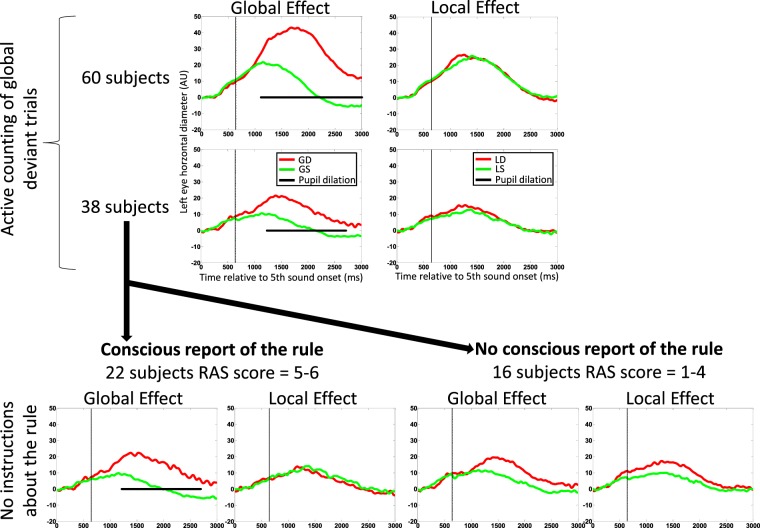
The pupil dilation global effect occurred only in participants aware of global regularity violations Using the same visual codes as in Fig. 3, a significant pupil dilation effect occurred in response to violation of global regularity, and not to violations of local regularity (artefact interpolation method; 2 upper panels). A subset of these 60 participants (N = 38) performed first a passive attentive version of the task during which they were not instructed about the structure of stimuli, and did not have to count global deviants, and were then submitted to a post-experimental regularity awareness score (RAS) to distinguish participants who consciously accessed to global regularity (RAS ≥ 5; N = 22) from those who did not (RAS < 5; N = 16). Finally they also performed an active counting version of the task. During the active counting task this subset of 38 participants showed the exact same pattern of results in the total group of 60 participants (two middle panels). In the passive attentive version of the task, only participants aware of global regularity showed a pupil dilation to global deviant stimuli.

The post-experiment interview enabled to distinguish between participants who explicitly reported the ‘global’ regularity of the stimuli from those who did not. We binned together participants with high RAS score (RAS ≥ 5; N = 22), and compared their pupillometry data with the ones who were not aware of the global structure of the stimuli and of the presence of global deviant trials (RAS < 5; N = 16). No ERPD difference was present between local standard and local deviant trials in both groups (see Fig. 4). Crucially, a pupil dilation effect was present in response to global deviant trials, - as compared to global standard trials -, only in the group of participants who were aware of the global regularity. The subgroup of 16 participants who failed to spontaneously access to the global regularity attribute, - and who did not present an ERPD global effect -, showed such a global effect in the active version of the task. We confirmed this ERPD difference between ‘aware’ and ‘unaware’ participants by calculating the interaction term of the ANOVA crossing awareness (aware/unaware) and global regularity (standard/deviant) (F(1,35) = 4.1; p = 0.05), on the mean pupil diameter across a temporal window showing a significant effect of global regularity in the group of 38 participants using the permutation procedure (1250–2700 ms).

At the individual level, the interpolation method enabled to analyze each of the 38 participants, and revealed a significant global effect (pupil dilation to GD trials) in 6/38 (15.8%), and a trend of such an effect (permutation corrected p-value: 0.05 < p ≤ 0.15) in 3 more participants. In contrast, only 1 participant showed a significant local effect, and 2 more participants showed a trend of such an effect.

We then focused on those 36 participants for whom we could record and analyze the pupillometry signal first without, and then with active counting instructions. Seven out of the nine participants showing a global effect (p ≤ 0.15) without instructions showed a global effect during the active counting task.

### Experiment 3

#### Behavior

The vast majority of the 16 participants easily counted the exact number of global deviant trials in each of the 2 experimental conditions (median error rate = 0; mean error-rate = 0.085 ± 0.4). The large standard deviation of error rates is due to one participant (T73), who counted the number of GS trials rather than the number of GD in Block 2.

#### Pupillometry

**Figure 5:**
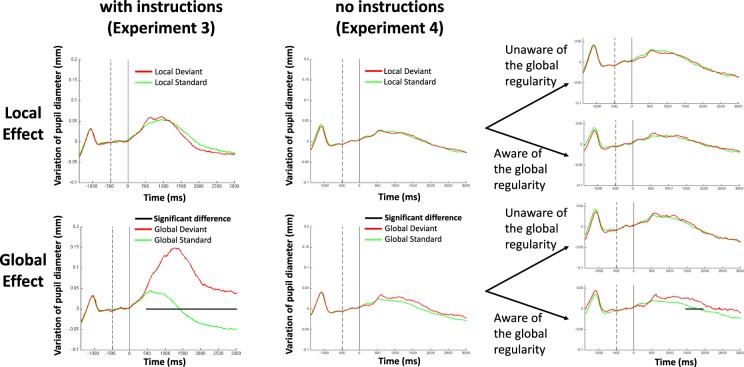
The pupil local and global effects in a phonemic version of the task Results of Experiments 3 and 4. Grand-averages of the variation of both pupil diameters (in mm) are plotted over time (in milliseconds) both for the local (upper panels) and global (lower panels) effects, with explicit counting instructions (Experiment 3, left panels) or without instruction (Experiment 4, right panels). While the event-related pupil response was undistinguishable between local standard (green curves) and local deviant (red curves) trials, a global effect was observed: a significant (black horizontal segments) pupil dilation was observed in response to global deviant trials. With explicit instructions, this effect began around 500 ms after the onset of the last syllable (time 0), peaked around 1.5 second and was sustained during more than 3 seconds. The effect was shorter and weaker without instructions, and only observed for those participants who were aware of the global regularity. The vertical dotted line indicates the beginning of the baseline period. The vertical solid line indicates the end of the baseline period, which corresponds to the temporal onset of the last syllable of each trial (time 0).

At the group level, no difference in pupil dilation was observed comparing local deviant to local standard trials, whereas larger pupil dilation was observed after global deviant trials than after global standard trials (p < 0.001; Fig. 5).

At the individual level, fourteen out of 16 participants (87.5%) showed a significant global effect (10 of these have p < 0.001). One additional participant (93%) showed a trend toward that effect at p = 0.12. The last participant showing no hint of a global effect is the participant who counted GS instead of GD in Block 2. In contrast, only 2 participants (12%) showed a local effect (p = 0.02 and p = 0.01). Six additional participants (50%) showed a trend towards the local effect (0.05 < p < 0.15).

### Experiment 4

We binned together participants who scored high on the RAS (RAS ≥ 5; N = 25) to compare their pupillometry data with the ones with lower RAS score (RAS < 5; N = 23). No ERPD difference was present between local standard and local deviant trials in either group, but a pupil dilation effect was present in response to global deviant trials as compared to global standard trials only in the group of participants who were aware of the global regularity (p < 0.05; Fig. 5).

As in Experiment 2, we confirmed this difference by calculating the interaction between rule awareness and global regularity within a temporal window in which the global effect was maximum (though not significant) across the 48 subjects (1467 ms to 1700 ms; threshold of t = 1.75 using the permutation procedure). In this time window, this critical interaction was found significant (F(1,46) = 3.97; p = 0.05).

Finally, at the individual level, 6 out of 48 participants (12.5%) showed a global effect (p < 0.05). Three of these showed larger pupil dilation for GS than for GD. Eight additional participants (29%) showed a trend towards a global effect (0.05 < P < 0.15). Two of these additional participants showed larger pupil dilation for GS than for GD.

Three out of 48 participants (6%) showed a local effect individually (one of these had major pupil dilation for LS). Four additional participants (15%) showed a trend towards the local effect (0.05 < p < 0.15). Three of these had major pupil dilation for LS.

## Discussion

In this study, we discovered a pupil dilation effect in response to violations of global regularity of trains of both tonal and phonemic auditory stimuli, whereas no such effect was present in response to violations of local regularity. Furthermore, we showed that this global effect was present exclusively in participants who were able to consciously report the existence of global regularities. This suggests that this pupil dilation effect could be a somatic marker of conscious access to global regularity violation. The similarity of pupil dilation in response to local standard and local deviant trials in both subgroups (report vs no report of global regularity) suggests that this result is not simply explained by a difference in level of vigilance. Similarly, our findings in the subgroups of participants who failed to spontaneously access to the global regularity and who did not show a pupil dilation effect also strengthens the links between conscious access and pupil dilation. These participants indeed showed a global effect when informed about this global regularity in the active counting task. Moreover, the existence of a global effect in the passive condition discards the possibility that pupil dilation simply reflected the effort of active counting of global deviant trials. These results were reproduced in two independent laboratories, with two distinct eye-trackers and pupillometry methods, and in two versions (tonal and phonemic) of the ‘local global’ paradigm. Note that in the phonemic version of the task we did not cross the 4 conditions within participants but across participants. However this did not prevent us to compute the local and global effect.

Furthermore, we showed that the global effect could be identified with satisfactory statistical significance at the individual level. The larger performance we observed in the phonemic version (93%) than in the tonal version (75%) of the active conditions (Exp.1 and Exp. 3) is probably related to the larger number of global deviant trials in the former (48 GD) than in the later (20 GD). This could be easily improved in future studies, and therefore highlights the potential use of our paradigms to study inter-individual differences or to determine individual level of consciousness.

Moreover, our results may lead to the general prediction that any conscious access may be accompanied by a pupil dilation. Indeed, a substantial set of results are already available regarding the neural and cognitive mechanisms at work during the ‘local global’ task we used. While violations of local regularities induce an early (~120 ms) and transient mismatch negativity (MMN) ERP effect restricted to auditory cortices (based on fMRI, SEEG, MEG and high-density EEG data^17,25,26^), violations of global regularities are associated to a late (>250 ms) and sustained P3b ERP effect widely distributed within a brain-scale fronto-parietal network, coherent with the global workspace model of conscious access^27–29^. In high-density EEG and MEG versions of the task, this P3b was observed exclusively in conscious participants who were aware of the violations of global regularity. Moreover, we previously showed that an ERP global effect is observed only in conscious controls and conscious patients, and in patients in a minimally conscious state^20^. The extremely rare patients clinically diagnosed as being in a non-conscious vegetative state with a global effect evolved clinically to a MCS a few days after they performed the task, suggesting they were misdiagnosed and actually conscious of global regularities^19^. Therefore, the triangulation of these previous findings with our current work linking subjective report of global regularity to pupil dilation, leads to the conclusion that pupil dilation is most probably contemporary of a P3b, and is a very likely somatic signature of conscious access. However, additional works are needed to confirm the specificity of this putative signature of conscious access^30^ by providing subjective reports at the single-trial level. Using paradigms such as the attentional blink^28^, or visual masking close to the threshold of conscious access^31,32^ one may validate that a pupil dilation effect is present exclusively for consciously reported stimuli.

Our results also suggest that interactions between brain activity and somatic markers could contribute to conscious perception by feeding a neural model of the self, as suggested by some experimental and theoretical studies^33–38^.

Finally, the tight link we identified between conscious access and pupil dilation encourages the current developments of pupillometry in cognitive science. Combined to our findings, the recent achievement of automated temporal deconvolution to reach high-temporal-resolution tracking of cognitive processes from the slow pupillary response^39^ paves the way to a dynamic probing of the stream of consciousness in controls, in clinical populations, as well as in preverbal infants. As we conjectured in the introduction, the design of original paradigms probing conscious access without interfering by questioning are now at our fingertips. The same approach can also be used to improve detection of conscious access to a stimulus or to a stimulus attribute (e.g.: regularity or semantics), - and therefore infer conscious state -, at bedside with cheap, non-invasive, relatively low-cost and non-constraining technology in non-communicating patients.

## Electronic supplementary material

supplementary information

## Data Availability

The experimental datasets generated and analyzed during the current study are available from the corresponding author on reasonable request.
