# Supplementary material for: Conscious processing of auditory regularities induces a pupil dilation
Source: Sci Rep. 2018 Oct 4;8:14819. doi: 10.1038/s41598-018-33202-7 (PMC6172267; doi:10.1038/s41598-018-33202-7)
Supplement: Supplementary file 1 — supplementary information [file 41598_2018_33202_MOESM1_ESM.pdf]

Supplementary Information

Conscious processing of auditory regularities induces a pupil dilation

Marion Quirins[1,2], Clémence Marois[1,2], Mélanie Valente[1,2,3], Magali Seassau[1,2], Nicolas Weiss[1,2,3,5,6,7,8], Imen El Karoui[1,2], Jean-Rémy Hochmann[9,10], Lionel Naccache[1,2,3,4,5,8]

1. INSERM, U 1127, F-75013, Paris, France
2. Institut du Cerveau et de la Moelle épinière, ICM, PICNIC Lab, F-75013, Paris, France
3. Sorbonne Universités, UPMC Univ Paris 06, Faculté de Médecine Pitié-Salpêtrière, Paris, France
4. AP-HP, Groupe hospitalier Pitié-Salpêtrière-Charles Foix, Department of Neurophysiology, Paris, France
5. AP-HP, Groupe hospitalier Pitié-Salpêtrière-Charles Foix, Department of Neurology, Paris, France
6. INSERM UMR_S 938, CDR Saint-Antoine Maladies métaboliques, biliaires et fibro-inflammatoires du foie, Paris, France
7. Institut de Cardiométabolisme et Nutrition, ICAN, Paris, France
8. Institut de neurosciences translationnelles IHU-A-ICM, Paris, France
9. CNRS, UMR 5304, Institut des Sciences Cognitives *Marc Jeannerod*, Bron, France
10. Université Claude Bernard Lyon 1, France

Corresponding author: Lionel Naccache

lionel.naccache@gmail.com

Key-Words: pupillometry, consciousness, conscious access, auditory, novelty, human, cognition

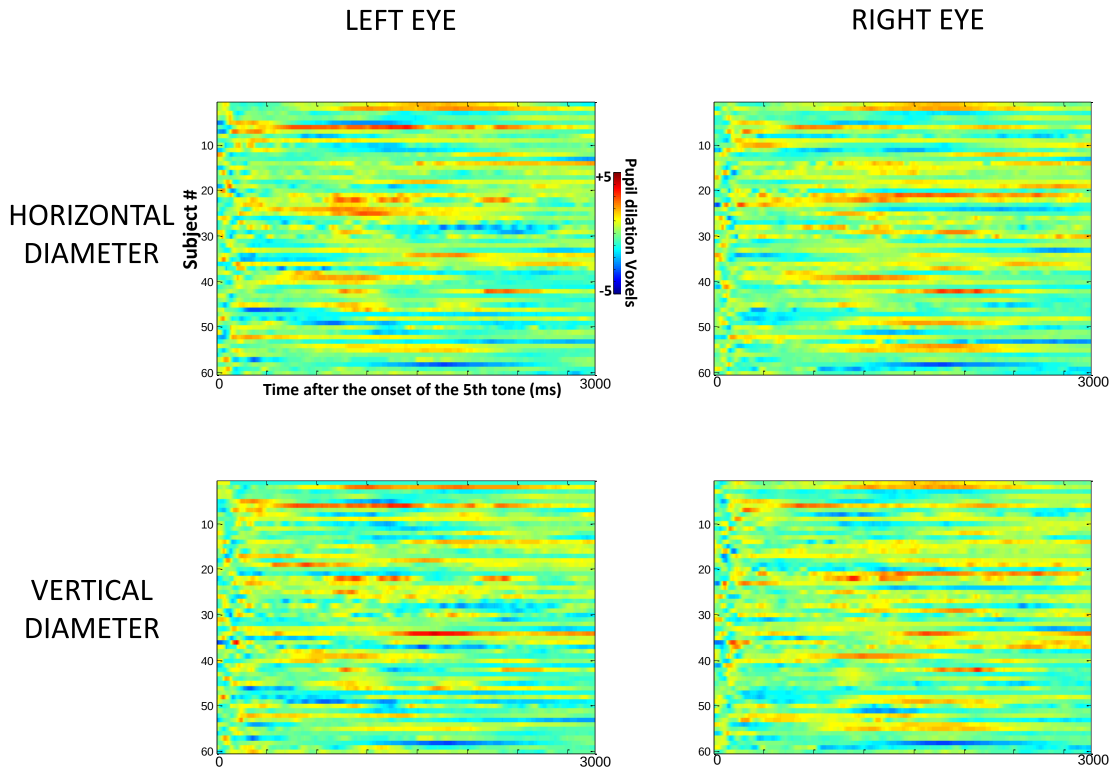

Figure S1: Averaged individual time-courses of ERPD in response to violations of local regularity during the active counting version of the 'local global' task

For each subject, the averaged ERPD in response to local standard trials was subtracted from the averaged ERPD in response to local deviant trials. Horizontal (upper panels) and vertical (lower panels) pupil diameters of left (left panels) and right (right panels) eyes are displayed for each of the 60 subjects. Local regularity did not affect pupil diameter (see Results). Note that results are similar for both eyes and diameters.

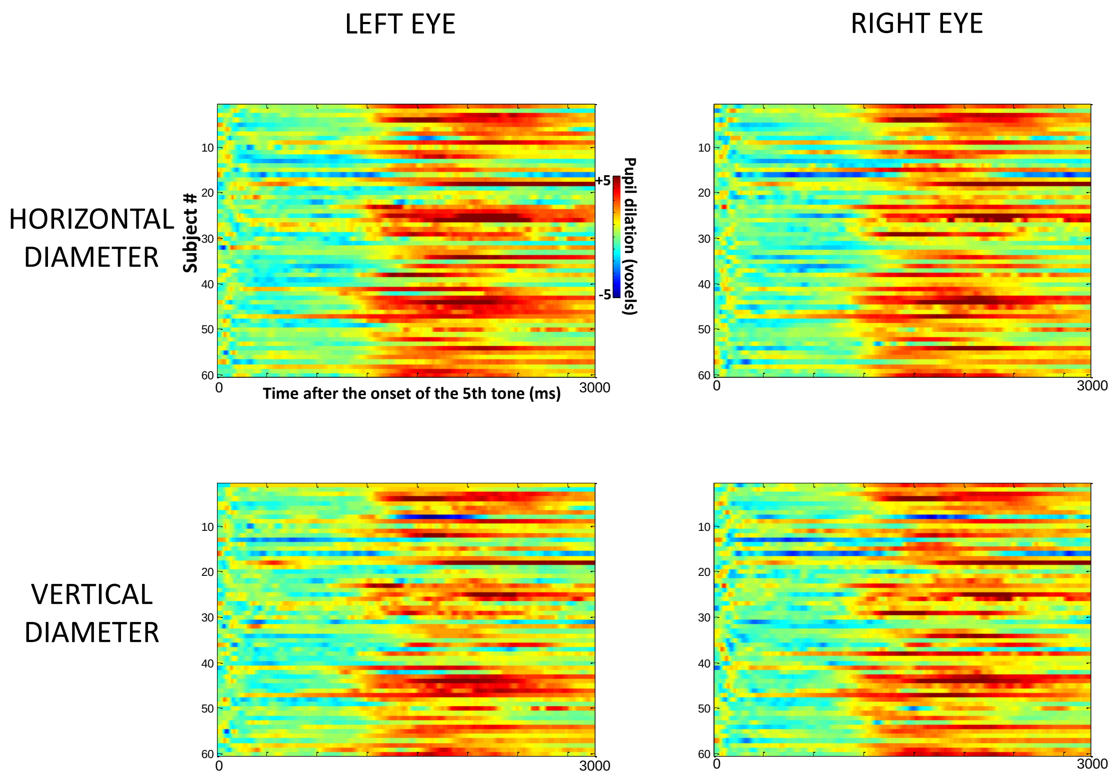

Figure S2: Averaged individual time-courses of ERPD in response to violations of global regularity during the active counting version of the 'local global' task

For each subject, the averaged ERPD in response to global standard trials was subtracted from the averaged ERPD in response to global deviant trials. Horizontal (upper panels) and vertical (lower panels) pupil diameters of left (left panels) and right (right panels) eyes are displayed for each of the 60 subjects. A pupil dilation to global deviant trials was observed and found significant in most individuals (see Results). Note that results are similar for both eyes and diameters.

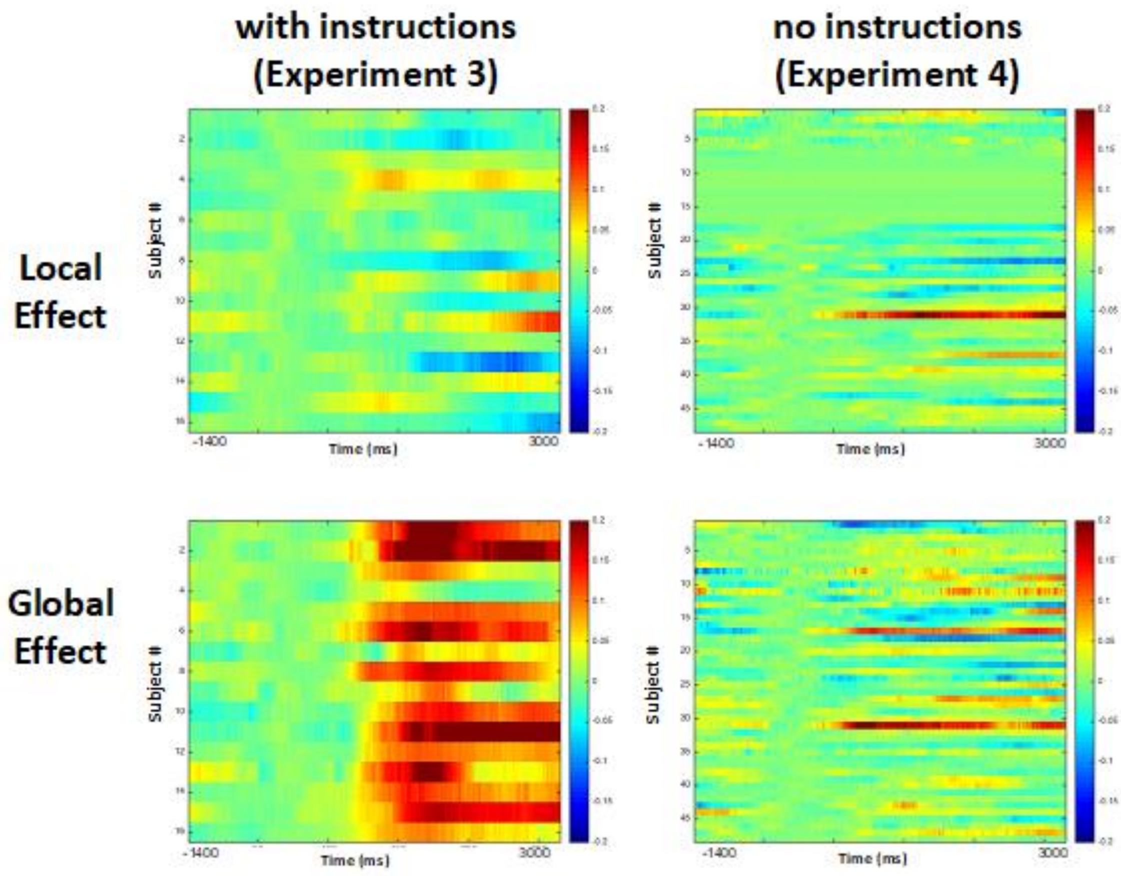

Figure S3: Averaged individual time-courses of ERPD in response to violations of local and global regularity during the active counting and passive attentive versions of the phonemic version of the ‘local global’ task

For each subject, the averaged global (bottom row) and local (top row) ERPD effects are shown (expressed in mm), both during the active counting version (left column) and the passive attentive version (right column) of the phonemic version of the task. A massive pupil dilation is observed in most individuals in response to violations of global regularity during the active counting condition.
